# Proteotranscriptomic Insights into the Venom Composition of the Wolf Spider *Lycosa tarantula*

**DOI:** 10.3390/toxins12080501

**Published:** 2020-08-05

**Authors:** Dominique Koua, Rosanna Mary, Anicet Ebou, Celia Barrachina, Khadija El Koulali, Guillaume Cazals, Pierre Charnet, Sebastien Dutertre

**Affiliations:** 1 Institut National Polytechnique Félix Houphouet-Boigny, BP 1093 Yamoussoukro, Cote D'Ivoire; koua.dominique@gmail.com (D.K.); anicet.ebou@gmail.com (A.E.); 2 Institut des Biomolécules Max Mousseron, UMR 5247, Université de Montpellier, CNRS, 34095 Montpellier, France; rosanna.mary@etu.umontpellier.fr (R.M.); guillaume.cazals@umontpellier.fr (G.C.); Pierre.CHARNET@cnrs.fr (P.C.); 3 MGX, Université de Montpellier, CNRS, INSERM, 34094 Montpellier, France; celia.barrachina@gmail.com; 4 BioCampus Montpellier, Université de Montpellier, CNRS, INSERM, 34094 Montpellier, France; Khadija.El-Koulali@fpp.cnrs.fr

**Keywords:** Spider, toxins, proteomics, transcriptomics, electrophysiology, cytolytic peptide

## Abstract

Spider venoms represent an original source of novel compounds with therapeutic and agrochemical potential. Whereas most of the research efforts have focused on large mygalomorph spiders, araneomorph spiders are equally promising but require more sensitive and sophisticated approaches given their limited size and reduced venom yield. Belonging to the latter group, the genus *Lycosa* (“wolf spiders”) contains many species widely distributed throughout the world. These spiders are ambush predators that do not build webs but instead rely strongly on their venom for prey capture. *Lycosa tarantula* is one of the largest species of wolf spider, but its venom composition is unknown. Using a combination of RNA sequencing of the venom glands and venom proteomics, we provide the first overview of the peptides and proteins produced by this iconic Mediterranean spider. Beside the typical small disulfide rich neurotoxins, several families of proteins were also identified, including cysteine-rich secretory proteins (CRISP) and Hyaluronidases. Proteomic analysis of the electrically stimulated venom validated 30 of these transcriptomic sequences, including nine putative neurotoxins and eight venom proteins. Interestingly, LC-MS venom profiles of manual versus electric stimulation, as well as female versus male, showed some marked differences in mass distribution. Finally, we also present some preliminary data on the biological activity of *L. tarantula* crude venom.

## 1. Introduction

Animal venoms consist of a complex mixture of bioactives, including small molecules, peptides, and proteins [1,2,3]. These natural libraries of compounds have evolved to target specific ion channels and receptors, and they are now actively being mined to discover new pharmacological probes but also potential drug and eco-friendly agrochemical candidates [4]. Among the venomous arthropods, spiders represent one of the most speciose invertebrate group, with more than 48,000 species described to date [1]. Spiders can be found in very diverse environments, having adapted to nearly all ground level niches and up to high in the canopy as well as under water [5]. One of the reasons for the evolutionary success of spiders comes from their ability to produce complex venom to protect themselves from predators (defense), and to facilitate prey capture (predation) [6,7]. Besides a sister clade known as the Mesothelae, spiders are broadly divided into the mygalomorphs (“ancient spiders”), the araneomorphs (“modern spiders”), the latter containing the vast majority of described species, (with approximately 39,000 species or >90% of all spiders).

Only a few spider species can be lethal to a fully grown adult human being, including the “terrible trio” made of the black widows (*Latrodectus sp.*), the wandering banana spider of South Americas (*Phoneutria nigriventer*), and the infamous Australian funnel-web spiders (*Atrax robustus* and *Hadronyche sp.*) [8]. Yet, any “black hairy spider” will almost instantaneously trigger uncontrollable fear in many people. As a result of their deadly potential, the venom of spiders has been the focus of many studies early on, notably to investigate the mode of action of the medically important venom components [8]. Remarkably, in the case of the Australian funnel-web spiders, it is the male that is particularly dangerous [9]. One explanation for this sexual dimorphism is that males need to leave the safety of their burrow to go find a female for mating, exposing themselves to predators and requiring “defensive toxins” rather than predatory toxins. These sex-driven venom intraspecific variations have since been demonstrated for other spider groups, including the ctenids and tetragnathids [10,11]. Other venom variations can be attributed to the method of collection [12]. Largely employed, electric stimulation of the venom glands produces higher yields but may differ from naturally produced venom. Therefore, caution is required when interpreting the results of electrically stimulated venom, since proteins and other components released from damaged secretory cells can alter the composition.

**Figure 1:**
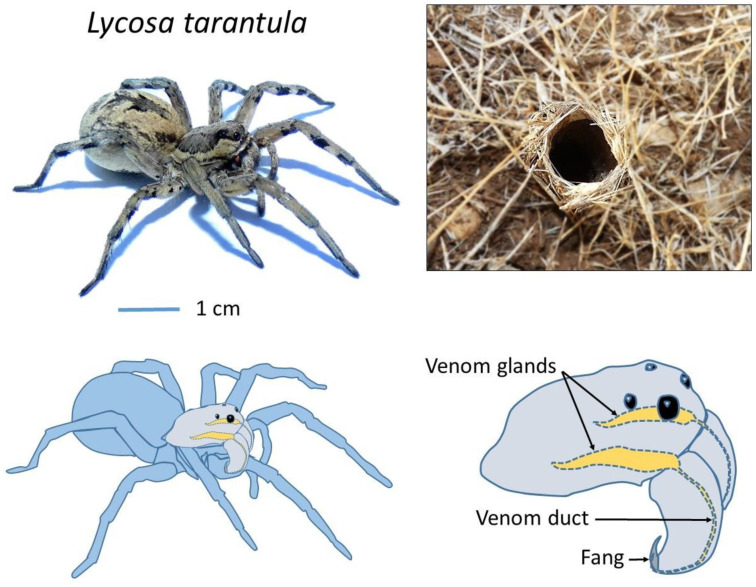
Adult female *Lycosa tarantula* specimen (**top left**), typical burrow entrance (**top right**) and localization of the venom glands (**bottom panels**).

Whereas many araneomorph spiders have evolved web-building skills to facilitate prey capture, some remain active hunters and rely on speed and fast acting venom to subdue their prey. From this latter group, the wolf spiders (Lycosidae) are widely distributed across the globe, with more than 2300 species described [13]. *Lycosa tarantula* is one of the largest representatives of the Lycosidae family in the Mediterranean basin [14], reaching a body length of 30 mm. It lives in burrows up to 40 cm deep, which end with a “turret” made of twigs, plant debris and small pebbles agglomerated with silk (Figure 1). From this burrow, the spider will ambush passing prey. It is found in southern Europe, including France, but also Italy, where this legendary spider was wrongly held responsible for the “tarantism”. A bite from this “tarantula” was supposed to cause a state of lethargy that may lead to death, and affected victims had to dance the “tarantella” if they were to survive [15]. It was later suggested that the true culprit was likely the local black widow (*Latrodectus tredecimguttatus*). Remarkably, although locally common in Mediterranean regions and relatively large, the venom of *L. tarantula* is still mostly unknown. Despite being considered harmless to humans, early experiments from naturalist Jean-Henri Fabre demonstrated that its venom can have deleterious effects on small vertebrates [16]. Indeed, a young sparrow and a mole bitten by an adult *L. tarantula* would both succumb to the effect of its venom in less than 48 h, suggesting that this species should be handled carefully.

Accordingly, the venom of other large Lycosidae, such as *L. singoriensis*, was shown to affect the physiology of vertebrates, including the contraction of a frog’s heart and the rat vas deferens [17]. However, the class of toxins responsible for these biological effects are unknown, and the full toxin repertoire produced by a *Lycosa* spider remains unclear. Although the venom gland transcriptomes of *L. singoriensis* [18] and *L. vittata* [19] have been obtained via traditional Sanger sequencing, the high throughput next generation sequencing technologies have only been applied to a single species to date, namely *Pardosa pseudoannulata* [20]. Yet, combination of venom gland transcriptomics and venom proteomic analysis has not been reported for any Lycosidae. Thus, we provide here our in-depth analysis of the venom composition of *L. tarantula* using an integrated proteotranscriptomic approach. Bioinformatic-based identification of putative toxin-like and protein sequences coupled to LC-MS/MS proteomic analysis of the electrically stimulated venom provide an important resource for a better understanding of the biology of *L. tarantula* and for the mining of novel pharmacological compounds of interest.

## 2. Results

### 2.1. Major Venom Peptide and Protein Families Retrieved from L. tarantula Venom Gland Transcriptome

**Figure 2:**
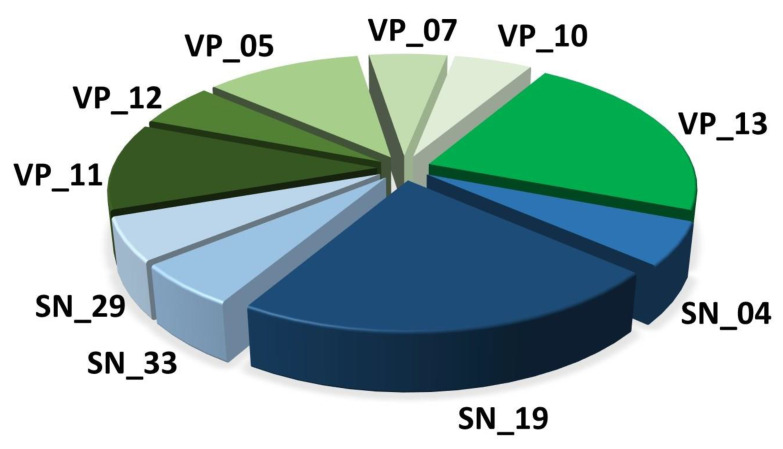
Representation of the 10 families of toxins (SN) and venom proteins (VP) from the venom gland transcriptome of *L. tarantula*.

To gain an unprecedented insight into the venom repertoire of *L. tarantula*, high throughput sequencing technology was employed to decipher the venom gland transcriptome. From the 28,793,065 raw Illumina reads obtained, a total of 389,316 contigs were assembled using Trinity version 2.1.1. All Contigs were annotated using an improved version of the previously published Ekenda Hidden Markov Models (HMM) library and the hmmcompete program [21]. Identified hits were classified into toxins family using Ekenda Hidden Markov Models (HMM) and the hmmcompete program. This classification is supported by an exhaustive set of 219 new profile hidden Markov models (HMMs) able to attribute a given peptide to its precise peptide type, family, and group [21]. Sequences were then annotated based on two consecutive BLASTs, one using a spider specific database and the second using the whole UniprotKB database (version of 2019_03). Signal Peptide and propeptide were predicted using respectively SignalP version 5.0 [22] and SpiderP tool available on the Arachnoserver webserver [23]. This automatic procedure takes advantage of the ability of HMMs to detect distantly related sequences and allowed to retrieve and to annotate a total of 18 putative sequences from 10 structural families including both venom proteins and neurotoxin-like typical peptides (including cysteine-rich, neurotoxin-like and linear peptides; Figure 2).

The sequences were deposited to GenBank (accession numbers: MT725462-MT725492). Among the 10 distinct structural families of venom related sequences, we found four families of spider neurotoxins (SN) and six families of venom proteins (VP). Classification as SN or VP were directly provided by the HMM-based annotation system used indicating sequence homology with spider neurotoxins and venom protein respectively [21] (Table 1). Although classified into venom proteins, VP-11 and VP-12 families contain short and disulfide rich peptides. Therefore, we included these two families in the next section on the description of disulfide rich peptide toxins.

#### 2.1.1. Disulfide Rich Peptide Toxins

In this section, the disulfide rich peptide toxins found in the transcriptome of *Lycosa tarantula* have been classified and named according to the nomenclature proposed by King et al. [24] The best matching sequence from BLAST search against UniprotKB database was aligned with each of the retrieved *L. tarantula* sequence for comparison. 

##### Family SN_04

**Figure 3:**
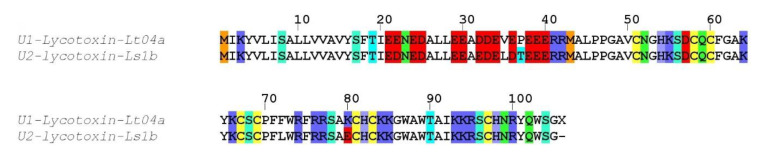
Sequence alignment between *L. tarantula* SN_04 sequence and the closest BLAST match. The letter X indicates a stop codon, confirming the precursor is complete. Color coding: yellow = cysteine, red = negatively charged residues, blue = positively charged residues, green = glutamine/asparagine, light green = serine, cyan = threonine, orange = methionine.

One sequence (U1-lycotoxin Lt04a) exhibited high identity (93%) with U2-lycotoxin-Ls1b from the related *Lycosa singoriensis* (Figure 3). Both signal peptides (1–18) were identical, whereas the propeptide contained five substitutions that were mostly conservative (e.g., E to D or V to L). The predicted mature toxin (cleavage after residue 42) contained eight cysteine residues arranged in a CX_6_CXCX_6_CXCX_11_CXCX_13_C pattern and showed only two substitutions compared to U2-lycotoxin-Ls1b, namely F72 to L and a charge inversion K80 to E. The biological activity of these toxins is unknown.

##### Family SN_19

**Figure 4:**
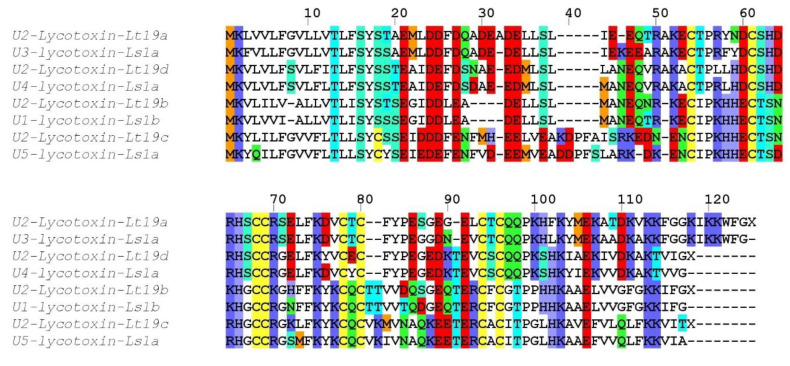
Sequence alignment between *L. tarantula* SN_19 sequences and the closest BLAST matches. Color coding: yellow = cysteine, red = negatively charged residues, blue = positively charged residues, green = glutamine/asparagine, light green = serine, cyan = threonine, orange = methionine.

Four distinct sequences grouped into SN_19 family showed high identity with several lycotoxins from *Lycosa singoriensis*. One sequence (U2-lycotoxin Lt19a) displayed 86% identity to U3-lycotoxin-Ls1a, a second sequence (U2-lycotoxin Lt19d), showed 91% identity to U4-lycotoxin-Ls1a, a third sequence (U2-lycotoxin Lt19b) 92% identity to U1-lycotoxin-Ls1b, and a fourth sequence (U2-lycotoxin Lt19c) 83% identity to U5-lycotoxin-Ls1a (Figure 4). Signal peptides and propeptides showed various degrees of conservation, but the predicted mature toxins all contained eight cysteine residues arranged in the same cysteine motif CX_6_CX_6_CCX_8_CXCX_10–13_CXC, justifying the grouping into one family. Interestingly, a rather long linear peptide was present at the C-terminal of all sequences. These peptides showed the typical features of the cytolytic, antimicrobial peptides (AMPs) described in Lycosidae [25], namely an amphipathic distribution of hydrophobic and charged residues, and may be cleaved off during the maturation process.

##### Family SN_29

**Figure 5:**
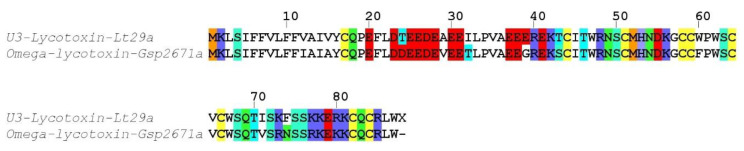
Sequence alignment between *L. tarantula* SN_29 sequence and the closest BLAST match. Color coding: yellow = cysteine, red = negatively charged residues, blue = positively charged residues, green = glutamine/asparagine, light green = serine, cyan = threonine, orange = methionine.

One sequence (U3-lycotoxin Lt29a) exhibited high identity (85%) with omega-lycotoxin-Gsp2671a from *Lycosa kazakhstanicus* (Figure 5). The signal peptides (1–17) and propeptides were highly similar and contained only few substitutions. The predicted mature toxin (cleavage after residue 40) contained eight cysteine residues arranged in a CX_6_CX_6_CCX_4_CXCX_15_CXC pattern and displayed seven substitutions compared to omega-lycotoxin-Gsp2671a. Omega-lycotoxin-Gsp2671a was shown to specifically modulate P-type Ca^2+^ channels [26].

##### Family SN_33

**Figure 6:**
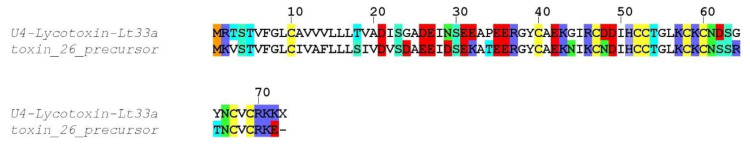
Sequence alignment between *L. tarantula* SN_33 sequence and the closest BLAST match. Color coding: yellow = cysteine, red = negatively charged residues, blue = positively charged residues, green = glutamine/asparagine, light green = serine, cyan = threonine, orange = methionine.

One sequence (U4-lycotoxin Lt33a) exhibited high identity (71%) with toxin 26 (accession MH754609.1) from the American wandering spider (*Cupiennius salei*; Figure 6). Despite both species being more distantly related, the signal peptides (1–25) were remarkably similar. After a relatively short propeptide, the predicted mature toxin (cleavage after residue 37) contained eight cysteine residues arranged in a CX_6_CX_4_CCX_4_CXCX_6_CXC pattern.

##### Family VP_12

**Figure 7:**
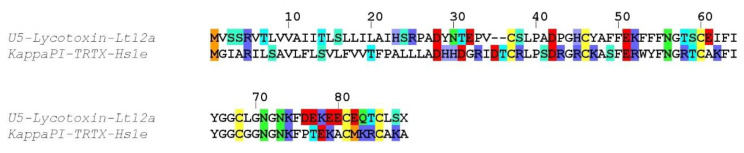
Sequence alignment between *L. tarantula* VP_12 sequence and the closest BLAST match. Color coding: yellow = cysteine, red = negatively charged residues, blue = positively charged residues, green = glutamine/asparagine, light green = serine, cyan = threonine, orange = methionine.

One sequence (U6-lycotoxin Lt12a) showed only limited identity (45%) to Kunitz-type kappaPI-theraphotoxin-Hs1e from the Chinese bird spider (*Haplopelma schmidti*; Figure 7). Despite a low sequence identity for the signal and propeptide regions, the six cysteine residues of the mature toxin were conserved and arranged in a CX_8_CX_13_CX_7_CX_12_CX_3_C motif.

##### Family VP_11

**Figure 8:**
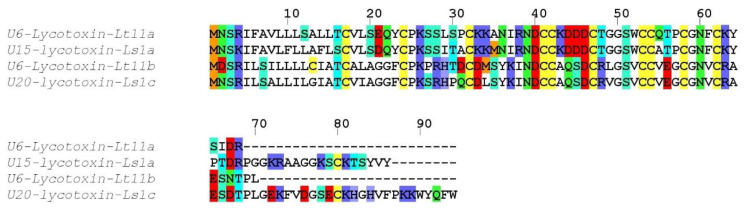
Sequence alignment between *L. tarantula* VP_11 sequences and the closest BLAST matches. Color coding: yellow = cysteine, red = negatively charged residues, blue = positively charged residues, green = glutamine/asparagine, light green = serine, cyan = threonine, orange = methionine.

Two incomplete sequences (U6-lycotoxin Lt11a and U6-lycotoxin Lt11b) exhibited high identity with U15-lycotoxin-Ls1a (81%) and with U20-lycotoxin-Ls1c (83%) from *Lycosa singoriensis* (Figure 8). Both signal peptides (1–20) were moderately conserved and no propeptide was predicted. The mature toxins (cleavage after residue 20) contained 10 cysteine residues arranged in a CX_7_CX_8_CCX_4_CX_5_CCX_3_CX_3_ CX_17_C pattern.

#### 2.1.2. Venom Proteins

In addition to the classic disulfide rich peptides, several sequences retrieved from *L. tarantula* venom gland transcriptome showed similarity with known venom proteins. These include hyaluronidase, angiotensin-converting enzyme, venom serine protease, and cysteine rich secretory protein (Table 1). The two partial hyaluronidase sequences identified showed 80–81% sequence identity to *Cupiennius salei* hyaluronidase. One complete sequence matched (52%) a putative angiotensin converting enzyme precursor from *Carcinus maenas*. One partial sequence showed high sequence identity (97%) to a putative processing quadruplet motif (PQM) protease precursor from *L. hispanica*, a sister species to *L. tarantula*. Finally, four partial sequences showed similarity to cysteine rich secretory proteins. From these, three sequences displayed high sequence identity to *L. singoriensis*’ venom allergen 5 proteins (81–91%), and one sequence shows moderate homology (62%) to a cysteine rich secretory protein 1 isoform a1 from *Cupiennius salei*.

### 2.2. Mass Spectrometry Analyses of L. tarantula Venom

**Figure 9:**
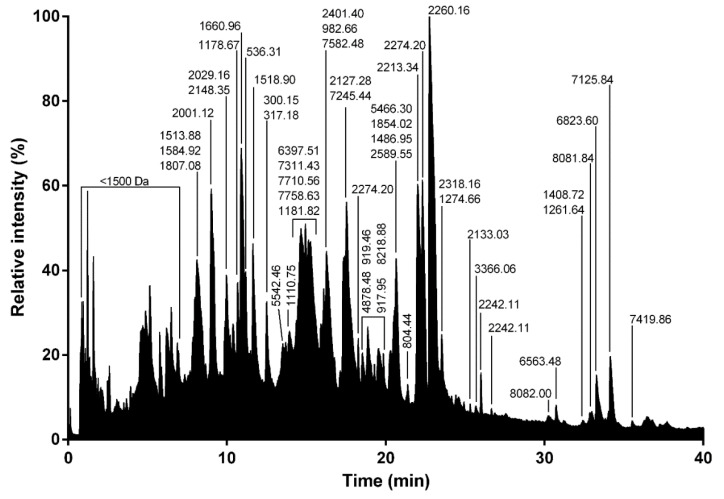
Total ion current trace of electrically stimulated (ES) *L. tarantula* venom. Major monoisotopic masses determined for each peak are indicated.

**Figure 10:**
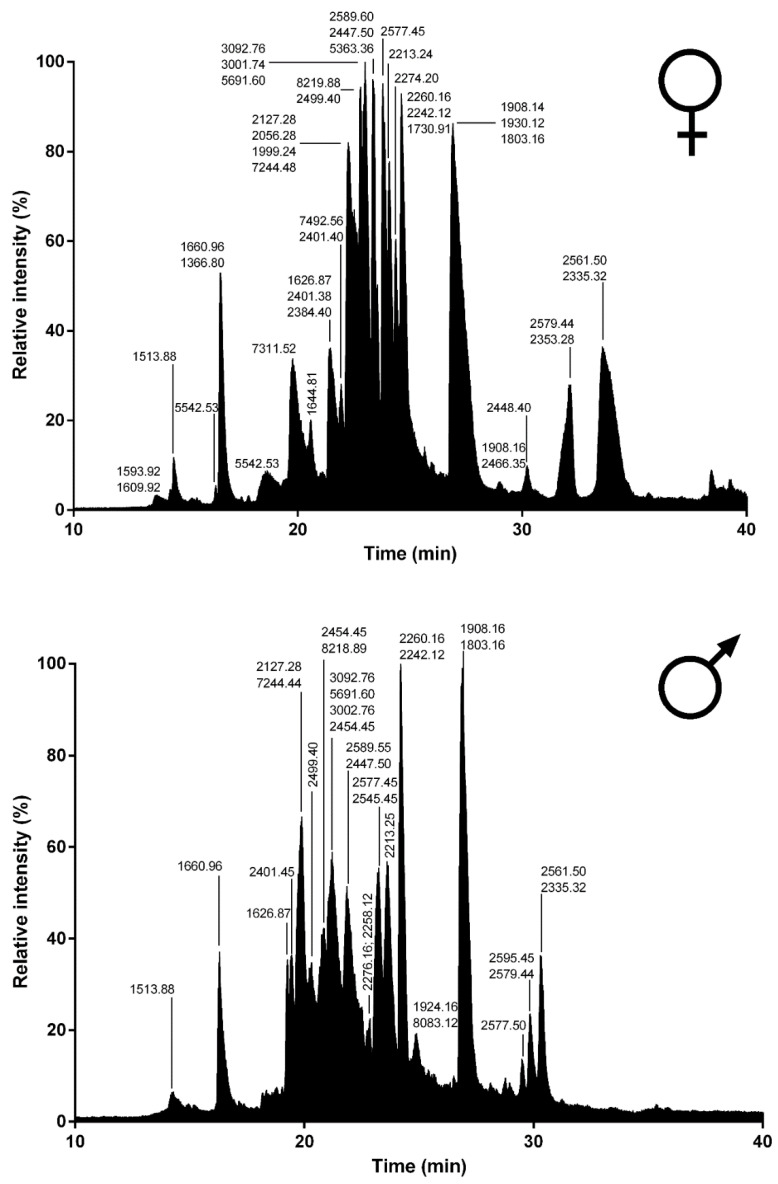
Total ion current traces of female and male *L. tarantula* venom. Representative traces of a single specimen (female, **top panel** and male, **bottom panel**). Major masses calculated for each peak are indicated.

To gain further insights into the venom composition of *L. tarantula*, mass spectrometry analyses were carried out, including comparative LC-MS of the electrically stimulated venom (Figure 9) and the manually collected venom from male and female specimens (Figure 10). In addition, a full proteomic (LC-MS/MS) analysis was performed on the electrically stimulated venom in order to test for the presence of some transcriptome-annotated venom peptides and proteins and validate their mature sequences.

#### 2.2.1. LC-MS of the Electrically Stimulated Venom

**Figure 11:**
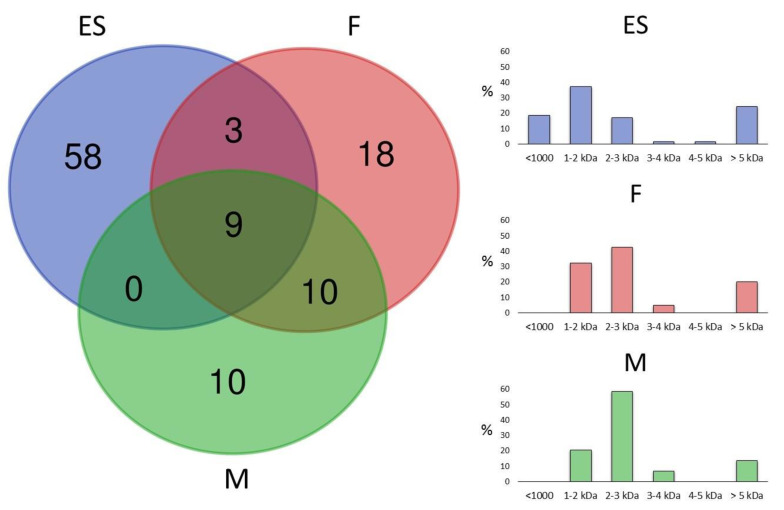
Venn diagram and mass distribution of electrically stimulated (ES), female (F), and male (M) *L. tarantula* venom. (**Left**) panel shows the overlap of masses between venom samples. (**Right**) panels show the mass distributions for each venom sample.

The venom from several specimens of *L. tarantula* was collected via electrostimulation and pooled. Approximatively 600 µg of venom was analyzed by LC-MS over 80 min. The overall total ion current (TIC) profile showed the highest complexity between 5 and 25 min (corresponding to 5–25% acetonitrile), where most of the ions were detected (Figure 9). The calculated monoisotopic masses for the dominant ions in each peak are reported on Figure 9, and the distribution shows a majority of masses <3 kDa, and then between 5 to 9 kDa (Figure 11). Interestingly, the top five most intense peaks correspond to small molecular weight compounds, between 1500–2500 Da (2260.16 Da, 1660.96 Da, 2274.2 Da, 2213.34 Da, and 2001.12 Da).

#### 2.2.2. LC-MS of Manually Stimulated Female vs. Male Venom

Although more convenient and producing higher yields, electrostimulation can damage secretory cells, resulting in the collected venom being “contaminated” with unwanted cellular proteins. Therefore, in an attempt to collect venom reflecting a more natural composition, we used a manual stimulation, where the spiders are aggravated and induced to bite into a plastic tubing. The resulting defensive venom droplets recovered from both male and female specimens were analyzed using LC-MS. The most striking difference with the electrically stimulated venom profile lies in the reduced complexity, especially in the early eluting compounds (no distinguishable peaks <10 min). Whereas the overall female vs. male profiles show obvious similarities in terms of complexity and peak intensities, the underlying differences appear more evident when considering the calculated masses and their overlap. Indeed, although the mass distribution showed a similar pattern, more than 50% of the masses detected in female venom were unique and not found in the male venom (Figure 11). Interestingly, in both venoms, one of the most intense ions corresponded to a mass of 1908.14 Da (together with 1803.16 Da), which appeared remarkably absent from the electrically stimulated venom.

#### 2.2.3. Proteomic Analysis of the Electrically Stimulated Venom

With the aim of identifying a maximum of the peptides and proteins present in the venom of *L. tarantula*, shotgun proteomics on a high-resolution mass spectrometer was performed on the more complex electrically stimulated venom. After reduction, alkylation, and trypsin digestion of the venom sample, the resulting peptides were fragmented, leading to the acquisition of 15,224 MS and 89,834 MS/MS scans, and further analyzed using PEAKS software (Bioinformatics solutions, Waterloo, ON, Canada). The search database was composed of our translated transcriptome, and a false discovery rate of 1% was applied. The results were filtrated in PEAKS Studio using stringent parameters, including peptide −10lgP ≥ 24.6, protein −10lgP ≥ 20, proteins unique peptides ≥2, and de novo average local confidence (ALC) score ≥80%. Under these conditions, 30 proteins were identified (Table S1). Among the validated sequences, the short neurotoxin-like peptides are well represented, with nine out the 10 sequences retrieved from the venom gland transcriptome that are validated. Overall, all disulfide rich peptide families were confirmed, except for the family VP_12 (Kunitz-type U5-lycotoxin-Lt12a). Next, the venom proteins were also well represented in the venom, with eight sequences validated, including all four CRISP (Venom allergen 5), two hyaluronidases, a putative PQM protease, and a putative angiotensin converting enzyme. Finally, some ubiquitous cellular proteins were identified, namely several heat shock proteins, cytochrome, elongation factor, arginine kinase, glyceraldehyde-3 phosphate dehydrogenase, actin as well as several sequences producing no significant match to known proteins.

### 2.3. Electrophysiology Assay of Crude L. tarantula Venom

**Figure 12:**
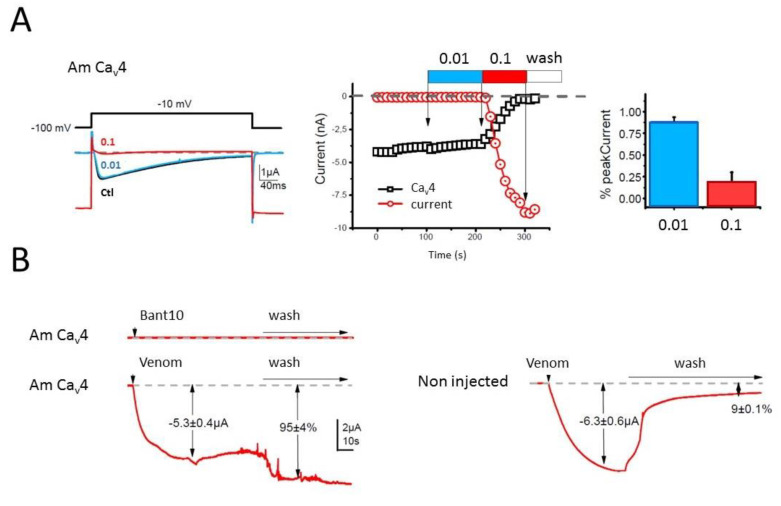
Biological effect of *L. tarantula* venom on *Apis mellifera* Cav_4_ channel expressed in oocytes. (**A**) Left panel shows the effect of *L. tarantula* venom on oocytes expressing the Ca_V_4 Ca^2+^ channel. Note the lack of effect at the 0.01 mg/mL dilution, and the strong increase in the holding current at the 0.1 mg/mL dilution. Middle panel represents the time course of the Ca^2+^ current and holding current amplitudes measured during the protocol shown in the left panel. The right panel shows the averaged effect on the peak Ca^2+^ current amplitude measured at the steady state or just before the wash. (**B**) Effect of *L. tarantula* venom on Ca_V_4-expressing (left) or non-injected oocytes (right) recorded on the holding current without any channel stimulation (constant holding potential of −100 Mv). Top left panel shows the holding current recorded continuously without depolarization in the Bant10 solution. The perfusion was stopped at the vertical arrowhead, a puff of 25 µL of Bant10 was applied in the recording chamber, without any effect, and the perfusion was started again at the horizontal arrow. Bottom left panel displays the same protocol applied but using 25 µL of the *L. tarantula* venom at 1 mg/mL instead of Bant10 solution. Note the big increase in the holding current and the lack of reversibility during the wash. Right panel shows the same protocol with a puff of *L. tarantula* venom at 1 mg/mL but applied to non-injected oocytes, with a similar increase in the holding current, indicating that this effect of the venom on the oocyte is independent of the expression of Ca_V_4.

The biological activity of crude (electrically stimulated) *Lycosa tarantula* venom was investigated using a two-electrode voltage clamp method on honeybee Ca_V_4 (DSC1) expressed in *Xenopus laevis* oocytes. Upon application of 0.01 mg/mL diluted venom, no significant effect was observed, but at 0.1 mg/mL, the increase in the leak current was so strong that the oocyte could not be properly clamped anymore, and thus value of the holding potential and the depolarization could not be maintained, preventing the adequate measurement of the Ca^2+^ current (Figure 12A). Suspecting that the venom strongly permeabilizes the oocyte membrane, the venom was also tested without depolarization on Ca_V_4–injected (*n* = 6) and non-injected (*n* = 7) oocytes. Indeed, application of 25 µL of venom (1 mg/mL) produced a similar increase in holding current, indicating that this effect is independent of the expression of Ca_V_4 (Figure 12B). However, in some oocytes (*n* = 2) a notable difference between injected and non-injected oocytes appeared upon washing of the venom. Whereas these two non-injected oocytes “recovered” from the leak (holding current amplitude back to smaller values), the other oocytes (six injected with honeybee Ca_V_4 and five non-injected) were unable to recover. This behavior prevents a clear detection of any Ca_V_4 channel blocker within the venom. Further deconvolution of *L. tarantula* venom will clearly be necessary to determine if it contains specific Ca_V_4 blockers.

## 3. Discussion

Spider venoms consist of complex mixtures of biologically active compounds that are for the most part gene encoded polypeptides and proteins. Therefore, combining venom gland transcriptomics with venom proteomics is a powerful method to accelerate the identification of full precursors and mature toxins for a better understanding of spider biology, venom-ecology relationships, and for the mining of useful pharmaceutical and agrochemical molecules. In this work, we used such proteotranscriptomics strategy to provide the first insights into the venom of one of the largest Lycosid spiders found in the Mediterranean region, *Lycosa tarantula*. Automated bioinformatics analyses followed by manual validation of the venom gland transcriptome revealed 18 distinct venom-related sequences classified into 10 structural families. The disulfide rich neurotoxin-like peptides comprised 10 sequences from six families, whereas the venom proteins were grouped into four distinct classes. Besides these sequences, proteomics investigations also revealed the presence of common cellular proteins, confirming that electrically stimulated venom includes contaminants. Indeed, the manually stimulated venom from both male and female specimens showed a less complex LC-MS profile and a different mass distribution compared to electrically stimulated venom. Interestingly, more than 50% of the masses detected in female venom were unique and not found in the male’s venom, suggesting that some intraspecific variations may be due to sex. Such intraspecific variations between male and female has already been reported in several species of spiders [27,28,29].

Whereas the biological activity of the neurotoxin-like peptides remains to be elucidated, our preliminary investigation of the crude venom on honeybee Ca_V_4 ion channel indicated the possible presence of selective blockers. However, further deconvolution of the crude venom will be necessary to uncover the peptides responsible for this activity, since the cytolytic activity present in the venom prevented accurate electrical measurement. Indeed, application of the crude venom to injected and non-injected oocytes induced a strong leak current, consistent with the cytolytic activity described for several other Lycosidae venoms. The molecular entities responsible for this cytolytic activity are known as antimicrobial peptides (AMPs), which are usually small, highly positively charged linear peptides adopting an amphipathic secondary structure in lipid membrane. Several such AMPs have been isolated and sequenced from Lycosidae venom [25]. Often, only the mature peptide sequences are available, not the full precursors, raising the question about the molecular origin of these AMPs. Interestingly, in the recently published high throughput sequencing of the venom gland of the Lycosidae *Pardosa pseudoannulata*, there is no mention of AMPs. However, a closer inspection of the reported sequences reveals that family A resembles the “inhibitory cysteine knot (ICK) + α-helix” modular toxin described from a Zodariidae spider, *Lachesana tarabaevi*. In these modular toxins, the C-terminal fragment synthesized separately was shown to possess membrane-binding activity consistent with a cytolytic effect [30]. These AMPs are often major components of the venom in Lycosidae, as seen with LyeTx I, a peptide isolated from *Lycosa erythrognatha* [31]. In our transcriptome, family SN_19 also displays the same architecture, with a N-terminal ICK motif and a C-terminal AMP-like sequence, and LC-MS of the venom shows a major contribution of peptides in the 1500–2500 Da range. Interestingly, the C-terminal peptide (QQPKSHKIAEKIVDKAKTVI) of U2-lycotoxin Lt19a has a mass (2260.32 Da) that corresponds to the major peak present in the venom (see Figure 9). The C-terminal peptides of the other SN_19 family sequences are also in the same mass range of 2000–2500 Da. Further work, including HPLC fractionation and purification steps, will be necessary to confirm this hypothesis.

Compared to the transcriptomes of other Lycosidae, such as *Lycosa singoriensis* or *Lycosa vittata*, our *Lycosa tarantula* transcriptome revealed a similar number of structural families, but fewer paralogs for each family [18,19]. However, it should be noted that in these studies, many of the reported paralogs were actually often single substitution sequence variants, and several of these substitutions were located in the propeptide, therefore producing identical mature toxin. We suspect that the assembly step of our Illumina reads eliminated the majority of these minor substitution variants that were otherwise picked up by the traditional Sanger sequencing technology used in these studies. However, we cannot exclude that additional neurotoxin-like sequences were missed. For instance, interrogation of the PEAKS “de novo” peptides that did not match any sequence from our transcriptome revealed a number of fragments that show high similarity to known neurotoxins, such as YPESGEGELCTCQQPK (75% U3-lycotoxin-Ls1h, *Lycosa singoriensis*), CTPLLHDCSHDR (92% U4-lycotoxin-Ls1b, *Lycosa singoriensis*), GCGFLDFNYPGDGR (93% Venom allergen 5, *Lycosa singoriensis*), and CCWPWSCVCWSQTLS (87% Omega-lycotoxin-Gsp2671e, *Alopecosa marikovskyi*). These unmatched yet high quality proteomic sequences may arise from the different specimens used for venom gland transcriptomics and venom proteomics.

In summary, we have reported here the first proteotranscriptomics analysis of *Lycosa tarantula* venom, including 18 distinct sequences of short neurotoxin-like peptides and venom proteins from 10 structural families. Future works should focus on the synthesis and pharmacological characterization of some of the neurotoxin-like peptides, as well as the cytolytic activity of some C-terminal fragments. Our data contribute to a treasure trove for the mining of useful pharmacological compounds.

## 4. Materials and Methods

### 4.1. Spiders, Venom Collection, and Venom Gland Dissection

Twelve specimens, including two mature males and 10 adult females of *Lycosa tarantula* were collected in the scrublands around Montpellier, France. These spiders (except males, caught wandering in open areas) were lured out of their burrow using a small stick wiggling around the entrance and caught into plastic jars. Specimens were then individually isolated in small boxes and maintained in the laboratory at room temperature. They were watered twice a week and fed once a week with commercially available mealworms.

To collect the venom from these specimens, two methods were used. First, a “manual stimulation” was applied similar to that described by Liu et al. [17], where each spider was presented with a piece of soft tubing (0.5 cm in diameter) and aggravated with tweezers to trigger a bite. Venom drops deposited on the tube were recovered using a pipette and diluted in distilled water. Secondly, electrostimulation was carried out on several specimens (*n* > 7) using an electric venom extractor based on the Arduino^®^ Mega 2560 board, specifically designed for the extraction of venom from arthropods and other small size animals [32].

Spiders were not fed for at least a week prior to the milking session. Specimens were anesthetized before milking (with 5% CO_2_). Chelicerae were stimulated by electrical impulses (3 to 7 V and approximately 0.5 to 2 A) discharged in a 2 s “working time” and 2 s of “rest time” steps. Released venom was collected from the tip of the fangs using a pipette and transferred to a 1.5 mL microcentrifuge tube containing approximately 20 μL of distilled water. Protein concentration of the venom samples were assessed using a nanophotometer N60 (Implen GmbH, München, Germany). Venom collected from individual spiders was pooled, freeze-dried and stored at −20 °C for subsequent use (proteomic characterization and electrophysiology).

To obtain the amount of mRNA required for the transcriptome sequencing, venom glands of six anesthetized adult female spiders were dissected on ice and placed in a 1.5 mL microcentrifuge tube containing 500 µL of lysis buffer. Next, the mRNA was extracted using a commercial kit (Magnetic mRNA isolation kit, Biolabs) following the manufacturer’s instructions. After extraction, the mRNA concentration was measured using a nanophotometer N60 (Implen GmbH, München, Germany).

### 4.2. Library prepaRation and Illumina Sequencing

RNA-Seq libraries were constructed with the Truseq stranded mRNA sample preparation (low throughput protocol) kit from Illumina (San Diego, CA, USA). Depending on the samples, 100 or 200 ng of mRNA was used for the construction of the libraries. Next, the mRNA was fragmented into small pieces using divalent cations under elevated temperature. The cleaved RNA fragments were copied into first strand cDNA using SuperScript II reverse transcriptase, Actinomycin D and random hexamer primers. The second strand cDNA was synthesized by replacing deoxythymidine triphosphate (dTTP) with deoxyuridine triphosphate (dUTP). These cDNA fragments have the addition of a single ‘A’ base and subsequent ligation of the adapter. The products are then purified and enriched with 15 cycles of PCR. The final cDNA libraries were validated with a Fragment Analyzer (Agilent Santa Clara, CA, USA) and quantified with a KAPA qPCR kit (Kapa Biosystems, Wilmington, MA, USA).

The transcriptome of *L. tarantula* was sequenced as part of a larger project comprising 15 other venom gland transcriptomes. On three sequencing lanes of V2 flowcells, the 16 libraries were pooled in equal proportions, denatured with NaOH and diluted to 18 pM before clustering. Cluster formation, primer hybridization and single-end read, 125 cycles sequencing were performed on cBot and HiSeq2500 (Illumina, San Diego, CA, USA) respectively.

Image analysis and base calling were performed using the HiSeq Control Software v.2.2.68 (Illumina, San Diego, CA, USA) and Real-Time Analysis component v.1.18.66.3 (Illumina, San Diego, CA, USA). Demultiplexing was performed using Illumina’s conversion software (bcl2fastq 2.18). The quality of the data was assessed using FastQC from the Babraham Institute v.0.11.5 and the Illumina software SAV (Sequencing Analysis Viewer) v. 2.1.8 (Illumina, San Diego, CA, USA). Potential contaminants were investigated with the FastQ Screen software from the Babraham Institute v.0.9.5.

### 4.3. Bioinformatics Sequence Analysis

Data issued from the sequencing platform were trimmed using the Trinity trimmomatic tool with default parameters. Reads were assembled using the Trinity software (version 2.1.1) [33]. Obtained contigs were translated in-silico into their six reading frames and annotated using the following procedure. An in-house database composed of all spider toxins from Arachnoserver, UniprotKB/SwissProt and Venomzone were created using makeblastdb of BLAST+ package after redundancy removal using CD-HIT [34,35] at the threshold of 1.00.

All Contigs were searched using an improved version of the previously published Ekenda Hidden Markov Models (HMM) library and the hmmcompete program [21].

All Contigs were submitted to a first BLAST step against this database to provide an annotated subset of the transcriptome. Annotated contigs were again BLASTed against the whole UniprotKB/SwissProt database to confirm the exactitude of obtained hits and remove false positive hits (BlastP against UniProtKB with e-threshold = 0.0001; matrix BLOSUM-62, non-filtering and gapped; UniProtKB/SwissProt 2019_03).

Spider toxin-related sequences were identified and classified into toxins family using Ekenda Hidden Markov Models (HMM) and the hmmcompete program. Signal Peptide and propeptide were predicted using respectively SignalP version 5.0 [22] and SpiderP [23] directly from the Arachnoserver web server at http://www.arachnoserver.org/spiderP.html. A final manual validation step was performed: multiple sequence alignments using MAFFT Version 7 [36], variant identification, and cleavage site validation. All peptide hits as well as their corresponding contigs sequences were further analyzed at nucleotide level to detect eventual mutations. Nucleotide sequence variants that obviously resulted from sequencing errors, assembly errors or frame shifts were excluded.

### 4.4. Proteomics

#### 4.4.1. Liquid Chromatography Coupled Mass Spectrometry (LC-MS)

RP-UPLC was operated on an Acquity H-Class ultrahigh performance liquid chromatography (UPLC) system (Waters, Corp., Milford, MA, United States) fitted with a UV detector (diode array detector) under the control of Waters MassLynx software (version 4.1). Separation of the *L. tarantula* venom (~600 μg) was achieved using a Kinetex C_18_ 100 Å column (2.1 × 150 mm, 3 µm) fitted with a pre-column. Elution was carried out using a gradient of 0–80% B (0.1% formic acid in acetonitrile) in 80 min. Samples eluting from the UPLC were introduced into the mass spectrometer at a flow rate of 500 µL/min. Acquisitions were carried out over the range 50 Da to 1800 Da m/z every 0.1 s on a Synapt-G2-S high-definition MS system (Waters, Corp., Milford, MA, United States). To obtain the molecular masses of the venom components eluting between 0 and 40 min, each peak from the total ion current (TIC) chromatogram was analyzed with Waters Mass Lynx software (version 4.1) (Waters, Milford, MA, USA).

#### 4.4.2. Shotgun Proteomics (LC-MS/MS)

Prior to shotgun proteomics, venom protein extracts were denatured, reduced, and alkylated. Briefly, each sample (~50 μg) was dissolved in 89 μL of triethylammonium bicarbonate (TEABC) 100 mM. One microliter of dithiothreitol (DTT) 1 M was added and incubation was performed for 30 min at 60 °C. A volume of 10 μL of iodoacetamide (IAA) 0.5 M was added (incubation for 30 min in the dark). Enzymatic digestion was performed by addition of 2 μg trypsin (Gold, Promega, Madison, WI, USA) in TEABC 100 mM and incubation overnight at 30 °C. After completing the digestion step, peptides were purified and concentrated using OMIX Tips C18 reverse-phase resin (Agilent Technologies Inc., Santa Clara, CA, USA) according to the manufacturer’s specifications. Peptides were dehydrated in a vacuum centrifuge.

Samples were then subjected to nano-flow liquid chromatography coupled to tandem mass spectrometry (NanoLC-MS/MS). Samples were resuspended in 20 μL formic acid (0.1%, buffer A) and 1 µL was loaded onto an analytical 25 cm reversed-phase column (75 mm inner diameter, Acclaim Pepmap 100^®^ C18, Thermo Fisher Scientific) and separated with an Ultimate 3000 RSLC system (Thermo Fisher Scientific, Waltham, MA, USA) coupled to a Q Exactive HF-X (Thermo Fisher Scientific, Waltham, MA, USA) via a nano-electrospray source, using a 123 min gradient of 6% to 40% of buffer B (80% ACN, 0.1% formic acid) and a flow rate of 300 nL/min. MS/MS analyses were performed in a data-dependent mode. Full scans (375–1500 m/z) were acquired in the Orbitrap mass analyzer (Thermo Fisher Scientific, Waltham, MA, USA) with a 60,000 resolution at 200 m/z. For the full scans, 3 × 10^6^ ions were accumulated within a maximum injection time of 60 ms and detected in the Orbitrap mass analyzer. The twelve most intense ions with charge states ≥2 were sequentially isolated to a target value of 1 × 10^5^ with a maximum injection time of 45 ms and fragmented by higher-energy collisional dissociation (HCD) in the collision cell (normalized collision energy of 28%) and detected in the Orbitrap mass analyzer at 30,000 resolution.

#### 4.4.3. Bioinformatic Integration of Proteomic and Transcriptomic Data

PEAKS Studio 8.5 software (Bioinformatics solutions, Waterloo, ON, Canada) was used to match MS/MS spectra obtained from proteomic analysis of *L. tarantula* venom. MS spectra were elucidated based on a personalized database resulting from assembled contigs translated into their six reading frames. Carbamidomethylation was set as fixed modification, while oxidation (M) was set as variable modifications, with maximum missed cleavages at 3 for trypsin digestion. Parent mass and fragment mass error tolerance were set at 5 ppm and 0.015 Da respectively. False discovery rate (FDR) of 1% and unique peptide ≥2 were used for filtering out inaccurate proteins. A −10lgP > 120 was used to estimate whether the detected proteins was identified by enough reliable peptides MS/MS spectra. In order to identify more relevant sequences, the Spider algorithm from PEAKS Studio software was used to find additional mutations or to correct the sequences. This algorithm corrects the sequences stored in transcriptomic database with de novo sequences based on MS/MS spectra, which allowed to identify post-translational modifications (PTMs) and mutations. Minimum ion intensity for mutation and PTMs was set to 5%, and ALC score ≥ 90 for de novo sequences leading to low precursor mass error in order to identify reliable PTM’s and potential mutations.

### 4.5. Electrophysiology

Ovaries were surgically removed from *Xenopus laevis* female, anesthetized using a 0.2% MS222 solution (Sigma Saint-Louis, MO, USA). After a first mechanical dissociation and extensive washing using the OR-2 solution (containing in mM: NaCl, 100; MgCl_2_, 2; KCl, 2; HEPES, 10), oocytes were isolated by approximately 2 h enzymatic dissociation using 1 mg/mL collagenase IA (Sigma Saint-Louis, MO, USA) dissolved in OR-2. Oocytes were then washed several times with OR-2 and selected in the survival medium (containing in mM: NaCl, 96; MgCl_2_, 2; KCl, 2; CaCl_2_, 1.8; HEPES, 10; pyruvic acid, 2.5; gentamycin, 50 ~μg/mL; neutralized at pH 7.2 using NaOH).

Oocytes injection was performed in the equatorial region by employing a home-made pneumatic injectory. *Xenopus* oocytes were microinjected with RNA corresponding to the AmCaV4 channel (1 µg/µL) of domestic honeybee, *Apis mellifera*. About 40 oocytes were injected with 1 µL of solution, and these injected oocytes were incubated at 18 °C in OR-2 solution for at least 24 h for 2–7 days at 19 °C under gentle agitation before recording. The survival medium was renewed daily.

Whole cell Ba^2+^ currents were recorded under two electrode voltage-clamp by employing the GeneClamp 500 amplifier (Axon Inst., Burlingame, CA, USA). Current and voltage electrodes were filled with a solution containing: KCl 3M; KOH. The bath-clamp head stage was connected to the bath using two agar bridges filled with 2% agar in 3M KCl, and the extracellular solution (physiological solution) was BANT10 (BaOH: 10 mM, TEAOH 20%: 12 mL, NMDG: 30 mM, CsOH: 2 mM, HEPES: 10 mM, pH = 7.2 with methane sulfonate). Injection of BAPTA (in mM: BAPTA-free acid (Sigma Saint-Louis, MO, USA), 100; CsOH, 10; HEPES, 10; pH 7.2) into oocytes was performed using a third microelectrode (in order to eliminate any Ca^2+^-activated Cl current). Under these conditions uncontaminated Ba^2+^ currents can be recorded. Ba^2+^ currents were elicited by series of depolarizing steps of 400 ms duration from a holding potential of −100 mV, to 10 mV every 10 s. Voltage-protocol and ionic currents were generated and recorded using the Clampex software (pClamp, ver 7.0, Axon Inst) (Molecular Devices, San Jose, CA, USA). Venom solution to be tested were prepared just prior to the experiment by adding the desired concentration in the BANT10 physiological solution. The different concentrations of venom (0.01, 0.1, and 1 µM) were then applied manually in a static bath using a pipette delivering a dose from about 20 μL.

The effect of administered venom was measured when steady state was reached (after about 1 to 2 min, i.e., 6–12 depolarizations) as a percentage of inhibition of the peak Ba^2+^ current amplitude recorded during a depolarizing pulse ranging of −100 to 10 mV. Data are presented as means ± S.E.M. from at least three oocytes. 

## Figures and Tables

**Table 1:** Classification of putative neurotoxins and venom proteins.

Family Name	Family Description	Number of Contigs
SN_04	Omega-agatoxin superfamily	1
SN_19	Spider toxin CsTx superfamily	4
SN_33	CsTx-26	1
SN_29	Omega-lycotoxin family	1
VP_11	Spider Whey Acidic Protein (WAP) family	2
VP_12	Venom Kunitz-type family	1
VP_05	Hyaluronidase	2
VP_07	Angiotensin-converting enzyme	1
VP_10	Venom Serine protease	1
VP_13	Cysteine rich secretory protein	4

## Supplementary Materials

The following are available online at https://www.mdpi.com/2072-6651/12/8/501/s1, Table S1: Proteomics results.
